# “Hot” vs “Cold” endoscopic stapes surgery: a matched case–control study

**DOI:** 10.1007/s00405-022-07739-3

**Published:** 2022-11-15

**Authors:** Giulia Molinari, Ignacio Javier Fernandez, Claudio Melchiorri, Marella Reale, Marco Bonali, Livio Presutti, Cecilia Lotto, Daniela Lucidi

**Affiliations:** 1 Department of Otolaryngology, Head and Neck Surgery, Policlinico S. Orsola-Malpighi, IRCCS Azienda Ospedaliero-Universitaria di Bologna, Via Massarenti, 9, Bologna, Italy; 2 Department of Specialistic, Diagnostic and Experimental Medicine, Alma Mater Studiorum University, Bologna, Italy; 3 Department of Otolaryngology, Head and Neck Surgery, Azienda Ospedaliero-Universitaria Policlinico di Modena, University of Modena and Reggio Emilia, Modena, Italy

**Keywords:** Stapes, Endoscopic stapes surgery, Microscopic stapes surgery, CO_2_ laser, Microdrill, Stapedotomy, Bone conduction, Air conduction, Air–bone gap

## Abstract

**Purpose:**

To compare hearing results and complication rates between two groups of patients operated on by endoscopic stapes surgery (ESS) for otosclerosis, either with CO_2_ fiber laser or microdrill.

**Methods:**

A case–control study was performed. All consecutive cases of CO_2_ fiber laser ESS operated at a single center during the period 2017–2020 (case group) were matched to a control group of patients operated by traditional technique, according to year of surgery, preoperative mean air–bone gap, sex and age. Audiological data from preoperative and postoperative examinations and complication rates were compared.

**Results:**

46 cases were included. Mean operative time was significantly longer in the laser cohort (65 min) than in the drill one (45 min) (*p* = 0.003). Similar results were found in the two groups regarding the mean postoperative BC-PTA. The high-frequency bone conduction resulted significantly higher in the laser group (*p* = 0.002), suggesting an overclosure effect in the laser group. Consistently, a significant improvement of the BC-PTA threshold at 2000 Hz postoperatively was found in the laser group (*p* = 0.034). The postoperative AC-PTA significantly improved in both groups at all frequencies (*p* < 0.05), except for the AC threshold at 8 kHz. Similar rates of complications were found in the two groups.

**Conclusion:**

This study is the first to compare hearing results and complications between CO_2_ fiber laser and microdrill in ESS. Our results demonstrated similar functional outcomes between the two groups, confirming ESS as safe and effective, regardless of the technique used.

## Introduction

Stapes surgery (SS) is the gold standard for treating otosclerosis, no effective medical treatments being available so far. Although traditionally SS has been performed using the microscope, lately the technological advancements have allowed the use of the endoscope as an alternative approach. A consistent number of papers showed comparable operative times, complication rates, and audiological outcomes between the two approaches [1–3].

Over the years stapedotomy fenestration techniques have undergone some modifications as well, switching from the use of microinstruments to microdrills, and eventually to lasers. Being “no touch” instruments, the latter combine high precision in reaching the target with the low risk of mobilization of the footplate, possibly reducing mechanical damage to the hearing and balance systems [4–6].

Among the available lasers for SS, the CO_2_ laser has gained popularity after its first application in 1989, in view of its positive tissue interaction properties and high absorption by water [7, 8]. Only recently a flexible CO_2_ laser with hollow-core photonic bandgap optical fibers was developed to reach the flexibility and mechanical robustness to be coupled with both rigid and flexible endoscopes.

Both CO_2_ laser and microdrill have been used extendedly during microscopic stapes surgery, with conflicting results regarding postoperative hearing function [4–9], but none of the published papers has investigated the application of CO_2_ laser in endoscopic stapedotomy so far.

The combination of the heat generated from the light source during endoscopic ear surgery (EES) with that conveyed by the laser may raise concern about a possible thermal damage to the inner ear with this technique. However, we hypothesized that fiber CO_2_ laser could be safely applied in a transcanal endoscopic setting, without an increased risk of thermal damage respect to the traditional technique. The aim of this study is to compare hearing results and complication rates between two groups of patients operated on by endoscopic SS for otosclerosis, either with CO_2_ fiber laser or microdrill.

## Methods

Patients who underwent endoscopic stapes surgery at the Department of Otolaryngology-Head and Neck Surgery of the University Hospital of Modena, Italy, in the period between 2017 and 2020 were retrospectively reviewed. The diagnosis of otosclerosis was formulated based on clinical history of progressive hearing loss, normal otoendoscopic findings, conductive hearing loss (gap − 30 dB in the range of 0.5–4 kHz) and the absence of cochleostapedial reflexes. Temporal bone computed tomography was not deemed necessary for surgical candidacy and the confirmation of otosclerosis was obtained testing the mobility of the ossicular chain and footplate intraoperatively in all cases.

All consecutive cases of CO_2_ fiber laser endoscopic stapes surgery were enrolled in the present study (case group) and matched to a control group of patients operated by traditional technique (microdrill) during the same period. The matching was performed according to year of surgery, preoperative mean air–bone gap (classified as < 40 dB and ≥ 40 dB), sex and age. Matching was performed by C.M. who was blinded to intraoperative findings and postoperative outcomes. Exclusion criteria were revision surgery and patients lost at clinical and audiological follow-up.

Data regarding pre- and postoperative hearing function, surgical procedure, operative time, intraoperative complications (chorda tympani section, gusher, footplate fracture) and postoperative complications were retrospectively collected from patients’ charts and follow-up visits. Facial nerve palsy, tinnitus, vertigo, sensorineural hearing loss (SNHL), infection, tympanic membrane perforation, perilymphatic fistula, taste disturbance, prosthesis dislocation or extrusion were considered among postoperative complications. Revision cases occurred during the mentioned time span were also noted.

All pure tone audiograms were carried out by the same team of audiologists and technicians. Audiological data from preoperative and postoperative examinations were compared. Postoperative assessment (otoendoscopy and PTA) was routinely carried out 1, 6 and 12 months after surgery. Comparison of audiological postoperative outcomes between the two groups was based on the one-year audiometric evaluation.

In particular, bone conduction (BC) and air conduction (AC) pure-tone average (PTA) were calculated, from preoperative and postoperative pure-tone audiometry, as the mean value among thresholds at 0.5, 1, 2, and 3 kHz frequencies, according to the committee on Hearing and Equilibrium of the American Academy of Otolaryngology–Head and Neck Surgery guidelines [10].

Mean air–bone gap (ABG) was calculated as the difference between AC-PTA and BC-PTA, while ABG closure was calculated as the difference between mean preoperative ABG and mean postoperative ABG, with a positive value indicating an improvement and a negative value indicating a worse post-stapedotomy gap. The AC gain was defined, as the difference between postoperative and preoperative AC-PTA. Stapedotomy was considered successful if the postoperative ABG fell within 10 dB.

High-frequency BC-PTA (HFBC) was assessed by averaging the BC threshold for frequencies 1, 2, and 4 kHz [11]. The preoperative to postoperative change in this value is a measure of SNHL. Positive values reflect improved BC levels (also referred to as overclosure), while negative values indicate high-frequency SNHL [11].

### Surgical techniques

A transcanal exclusive endoscopic approach was performed using a 3-mm diameter, 14-cm length, 0° operating rigid endoscope, under general anesthesia, as elsewhere reported [12]. Xenon light intensity was kept at 50% of the maximal power, as routinely done for any EES procedure at our Institution.

In the case group, the surgical procedure was performed with the assistance of the AcuPulse DUO CO_2_ laser system coupled with OtoLase™ fiber and handpieces (Lumenis Ltd., Yokneam, Israel). This CO_2_ laser has an infrared wave 10.6 micron, and was routinely set in fiber mode, single pulse modality with shot of 0.2 s and 2 W power for stapedial tendon and posterior crus sectioning, while shot of 0.05–0.1 s and 1 W power for stapes fenestration. The procedure consisted in laser stapedial tendon sectioning, laser posterior crurotomy, disarticulation of the incudo-stapedial joint, stapes suprastructure removal and laser stapedotomy with multi-shot technique. The final opening of the footplate was achieved through manual perforator or Fisch microhook, to reduce the risk of thermal damage to the inner ear.

In the control group, the Skeeter drill (Medtronic, Jacksonville, USA) with a diamond burr was used for posterior crurotomy and platinotomy. Two surgical strategies were adopted in this group, as previously described, based on anatomical configuration and surgeon’s preference:

standard surgical steps: disarticulation of the incudo-stapedial joint (ISJ), section of the stapedial tendon, drilling of the posterior crus, drilling or fracture of the anterior crus.

Partial reversal surgical steps: sectioning of the stapedial tendon by Bellucci’s scissors, drilling of the posterior crus of the stapes, disarticulation of the ISJ and fracturing of the anterior crus.

A platinum/polytetrafluoroethylene (Spiggle & Theis, Overath, Germany) or platinum/fluoroplastic (Richards, Olympus, USA) prosthesis was inserted into the platinotomy hole and the hook crimped on the long process of the incus using crimping forceps in both groups. Blood-soaked Gelfoam pledgets were put around the prosthesis, the TMF repositioned and the external ear canal packed with resorbable pledgets. All surgeries were performed by the senior author, with more than 20 years of experience in EES (L.P.)

Surgical time was recorded as the time between first incision of the ear canal and packing.

### Statistical analysis

The statistical analysis was performed using SPSS for Windows (IBM SPSS Statistics, Chicago, USA). Continuous variables were expressed as mean ± standard deviation (SD). Comparisons between groups were performed by Pearson’s chi-square or Fischer exact test for discrete variables, as appropriate. Student’s *t* test was used for continuous variables with normal distribution, while Mann–Whitney *U* Test was adopted for continuous variables without a normal distribution. The strength of the correlation between the parameters was obtained by Spearman’s rank correlation test. The results were considered as significant for *p* values < 0.05 with a confidence interval of 95%.

This research has been conducted in full accordance with ethical principles, including the World Medical Association Declaration of Helsinki. For this kind of retrospective investigation, the Ethical Committee of the University Hospital of Modena does not perform a formal ethical assessment.

## Results

Overall, 46 cases of endoscopic stapes surgery were included in this study, each group consisting of 23 patients. Six males and 17 females were present in each group (M:F = 1:3.4). Left-to-right ear ratio was 1:1.5. The overall mean age at surgery was 49.7–10.7 years (49.1 for case group vs 50.3 for control group, *p* = 0.707). Mean operative time was significantly longer in the laser cohort (65 min) than in the drill one (45 min) (*p* = 0.003).

Audiometric data are summarized in Table 1. Regarding preoperative hearing function, the two groups did not show statistically significant variations, both in the mean preoperative BC-PTA and AC-PTA. In the postoperative follow-up, similar results were found in the two groups regarding mean postoperative BC-PTA.

**Table 1:** Preoperative and postoperative audiometric data in the case and control group

	Overall	CO_2_ laser group	Drill group	*p* value (CO_2_ vs Drill)
**Preoperative**				
Mean BC-PTA	25.5 dB	27.6 dB	23.3 dB	0.211
Mean AC-PTA	60.4 dB	61.6 dB	59.3 dB	0.53
Mean ABG	34.9 dB	33.9 dB	35.9 dB	0.443
**Postoperative**				
Mean BC-PTA	22.4 dB	21.9 dB	22.9 dB	0.759
Mean AC-PTA	30 dB	29.1 dB	31 dB	0.608
Mean ABG	7.6 dB	7.2 dB	8.1 dB	0.54
ABG gain	77.8%	78.2%	77.4%	0.847
HFBC	3.1	5.8	0.5	0.002*

*BC* bone conduction, *PTA* pure-tone average, *AC* air conduction, *ABG* air–bone gap, *HFBC* high-frequency bone conduction

*Significant *p* value

The HFBC resulted significantly higher in the laser group vs the drill one (5.8 dB vs 0.4 dB, *p* = 0.002), suggesting an overclosure effect in the laser group. Consistently, when analysing the single BC thresholds, a significant improvement of the BC-PTA threshold at 2000 Hz postoperatively was found in the CO_2_ laser group (*p* = 0.034), as shown in Table 2.

**Table 2:** Comparison of single bone-conduction and air-conduction threshold in the CO_2_ laser and drill groups

	CO_2_ laser group					Drill group						
	Preop		Postop			Preop		Postop				
kHz	dB	SD	dB	SD	*p *value	dB	SD	dB	SD	*p* value	Delta	*p *value (Delta comparison)
BC-0.5	18.9	10	16.7	10.1	0.466	16.3	7.3	15.6	5.9	0.74	– 0.65	0.358
BC-1	26.5	12.5	22	11.9	0.213	18.5	8.5	19.1	10.9	0.822	0.65	0.009*
BC-2	34.3	15.4	24.8	14.2	0.034*	29.5	16	28.5	15.8	0.818	– 1.08	0.001*
BC-3	30.9	14.9	24	13.3	0.108	29	14.2	28.3	13.7	0.855	– 0.76	0.001*
BC-4	26.7	15.6	23.5	13.3	0.451	28.5	13.9	28.3	13.7	0.958	– 0.21	0.051
AC-0.5	63	12.1	25.6	11.1	< 0.001*	60	9.5	25.2	9.4	< 0.001*	– 34.78	0.357
AC-1	65	13.9	30	12.8	< 0.001*	58.7	7.9	28.7	14.5	< 0.001*	– 30	0.147
AC-2	59.1	17	32.2	15.4	< 0.001*	58.7	15.2	34	15.9	< 0.001*	– 24.7	0.051
AC-3	59.1	17.4	32.2	15.1	< 0.001*	59.8	17	36.2	15	< 0.001*	– 23.58	0.219
AC-4	58.7	21.1	35.2	16.7	< 0.001*	60.9	20.6	39.8	18.4	0.001*	– 21.08	0.504
AC-6	60.1	21.2	43	17.7	< 0.001*	59.5	22.3	45.8	19.2	0.0026*	– 13.8	0.370
AC-8	61.5	23.3	50.9	21.4	0.106	58.3	13.7	51.7	21.6	0.330	– 6.52	0.416

*preop* preoperative, *postop* postoperative, *kHz* kilohertz, *dB* decibel, *SD* standard deviation, *BC* bone conduction, *AC* air conduction

*Significant *p* value

Overall, the mean ABG improved in both laser and drill groups (7.2 dB vs 8.1 dB, respectively), and the ABG gain was similar (78.2% vs 77.4%), with a p value of 0.847.

**Fig. 1:**
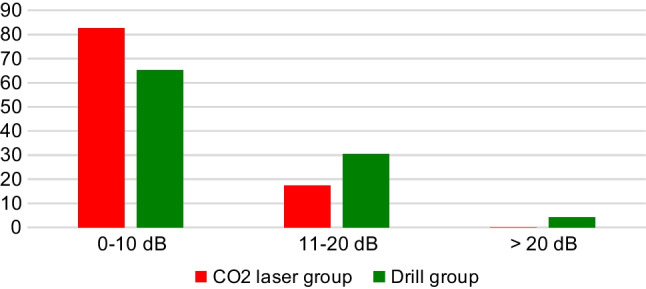
Distribution of postoperative mean air–bone gap in CO_2_ laser and drill groups, respectively (*p* = 0.318)

Specifically, in patients who underwent laser CO_2_ surgery, an ABG closure within 10 dB was found in 19 patients (82.6%) and a reduction G between 11 and 20 dB was found in 4 patients (17.4%). None of these patients had a residual ABG over 20 dB. Considering the cases operated by microdrill, there was a closure of ABG within 10 dB in 15 patients (65.3%), a reduction between 11 and 20 dB in 7 patients (30.4%) and a residual ABG over 20 dB only in 1 patient (4.3%) **(**Fig. 1). These differences, however, were not statistically different (*p* = 0.318).

Regarding the postoperative AC-PTA, it was significantly improved in both groups at all the frequencies (p < 0.05), except for the AC threshold at 8 kHz, which did not significantly improve in either group (*p* > 0.05; Table 2).

None of the patients had undergone revision surgery by the time of last follow-up.

Intraoperatively the chorda tympani was preserved in all but one case (control group) in which it was accidentally sectioned. No case of gusher, footplate fracture, facial nerve palsy, membrane perforation or infection occurred. In the CO_2_ group, vertigo and/or nystagmus occurred in 2 patients vs in the drill group they were limited to 1 patient only. Regarding taste disturbance, 1 patient from each group developed persistent dysgeusia, and 1 patient from the CO_2_ cohort complained of temporary taste changes. While SNHL did not occur in any of the groups, tinnitus was reported in 3 patients (13%) from the drill cohort. At last follow-up, none of the patients had prosthesis dislocation or extrusion.

## Discussion

Results of our study confirm that endoscopic SS for otosclerosis is safe and has favourable hearing outcomes. Postoperative ABG and ABG gain, as well as the distribution of postoperative ABG according to the degree of residual gap, were similar between the two groups, showing improved hearing after endoscopic SS regardless of the stapedotomy technique.

### Safety in endoscopic laser stapes surgery

One of the main concerns regarding the safety of the use of lasers in ESS, is associated with the risk of thermal injury of the inner ear and facial nerve. The effects of combining two heat sources (the light source of the endoscope and the laser itself) inside the middle ear should be investigated, especially when dealing with stapes surgery, where the laser is used directly on the footplate and the goal of surgery is to improve hearing function [23].

In the past, some reports on the use of the endoscope in the middle ear have investigated the effect of the use of the light source of the endoscope on the temperature increase inside the middle ear. On a guinea pig model, Dundar et al. demonstrated that rigid endoscopes cause a temperature increase in the tympanic cavity, regardless of their diameter, when used with xenon and halogen light sources. They concluded that this heat could easily be transmitted to the cochlea by the perilymph, after stapedotomy, resulting in neurosensorial damage [13]. Kozin et al. confirmed these data on cadaveric human temporal bones, showing that temperature elevation at the round window increases with proximity of the endoscope tip, but they also demonstrated that the frequent removal of the light source and the use of suction result in rapid cooling [14]. According to a Japanese study on 3D-printed temporal bone simulators, temperatures produced by an endoscope with LED light source are safe with a 2.7 mm optic coupled to a light intensity of 40% of the maximal output [15].

Multiple clinical studies on transcanal endoscopic SS have followed, showing no clinically relevant complications related to heat damage, nor on the facial nerve or the hearing and vestibular function [16–20]. This may be the result of the suggested technical strategies of half-intensity light source use, frequent aspiration of fluids and repeated cleansing of the endoscopic tip [21, 22].

So far, only one study investigated CO_2_-laser endoscopic stapedotomy in 4 patients, without reporting hearing outcomes [21], while other researchers reported on patients operated by endoscopic laser-assisted stapedotomy, without specifying the type of laser [24, 25].

To the best of the authors’ knowledge, our study is the first to compare the use of CO_2_ laser versus microdrill in endoscopic SS and to investigate the effects of inner ear function, showing a similar safety profile for both groups, measured by BC variations.

### Comparison of hearing outcomes between groups

According to our results, considering postoperative mean BC-PTA, a conservative effect was found in both groups. However, when considering HFBC, a significantly higher overclosure rate was found in the CO_2_ laser group compared to the drill group. Overclosure is an interesting occurrence after stapes surgery, according to which there is an improvement in bone conduction values and this has been related to Carhart phenomenon, an increase of the BC at 2–4 kHz after stapedotomy [11].

In our study, not only did the 2 kHz BC-PTA significantly improve in the CO_2_ laser group, but also the p-value of the comparison between microdrill and CO_2_ laser on the same frequency was significant (*p* = 0.001). Interestingly, also both 1 kHz and 3 kHz mean BC postoperative thresholds showed an increase in the CO_2_ laser group (despite not significant, *p* = 0.213 and 0.108 respectively). When compared to the results from the same frequencies in the microdrill group, the *p* value reached statistical significance (*p* = 0.009 and *p* = 0.001 respectively). A worsen 1 kHz BC value was found after microdrill stapedotomy, with no significant p-value.

Other authors have reported similar data in microscopic cohorts comparing microdrill to CO_2_ fenestration [26], while Altamani and colleagues reported that in large cohort, despite hearing improvement at 2 kHz in both groups (Carhart phenomenon), more hearing damage was observed in CO2 laser fenestration technique at 4 kHz [11]. Another large series from Somers et al. showed no statistically significant difference between the two techniques with regard to overclosure [27].

Data from our study not only support the absence of additive thermal damage from the laser on the inner ear, but also an advantage of the laser over the drill on the improvement of the postoperative BC threshold. This evidence is of further relevance if we consider that we used a “multiple-shot” technique to achieve stapedotomy in our series, as opposed to what is routinely performed in microscopic stapedotomy through modern microscope-mounted manipulators paired with scanner systems. Given the power limitations of the hollow fiber through which the laser is conveyed on the middle ear structures, several laser shots are necessary in the endoscopic setting to progressively thin the footplate and open the vestibule. “One-shot stapedotomy” advocates agree on a reduced exposure of the patients to the danger of repeated shots on an already opened vestibule, and a more regular shape and predictable size of the stapedotomy hole [11, 28]. According to previous reports, minimal energy necessary to open the footplate without significant hearing damage is achieved with one shot of focussed laser beam with scanner [29, 30].

It could be hypothesized that our favourable results may be related to the pulse duration with extremely low power, and completion of the fenestration with cold instruments. Nevertheless, our auditory outcomes from multi-shot technique with fiber CO_2_ laser in the endoscopic setting deserve further comparison with larger scale cohorts.

### Advantages and disadvantages of CO_2_ laser-assisted endoscopic stapedotomy

Among advantages of CO_2_ fiber laser in EES, Cohen underlined that compared to argon and KTP lasers, which require protective glasses with orange tinted lenses, the CO_2_ laser can be used with clear lenses [31]. Additionally, the hollow-fiber CO_2_ laser system does not have an aiming beam, but a helium jet which passes through the fiber and creates a restrained visible mark on the target tissue, without obscuring the endoscopic view [31].

Other advantages of CO_2_ laser are similar to those reported for microscopic stapes surgery literature. In particular, the main advantage is the reduction of mechanical forces imparted to the stapes during crurotomy and stapedotomy, thus minimizing the likelihood of displacing or fracturing the footplate during those surgical steps.

Among the disadvantages, the use of CO_2_ laser correlated with significantly longer operative times in our cohort. The reason may be looked for in technical issues during setting up the laser, as similarly reported by De Vito et al. in their microscopic experience [9]. Moreover, economical aspects should also be considered. According to an American multicentric report, costs associated with CO_2_ laser utilization were found significantly higher than with KTP laser, both in terms of surgical supply alone ($852.60 vs $230.55) and of total encounter cost ($4645.43 for CO_2_ laser vs $2903.00 for KTP laser vs $2932.47 for laser-free surgery).

### Strengths and limitations

Despite the limited number of patients included, the matching process on the preoperative hearing function was accurate, as confirmed by the lack of statistically significant differences between pre and postoperative BC-PTA and AC-PTA values between the groups. It should be underlined that the two cohorts of patients were operated in the same time-span with both of the techniques, and thus a possible “learning curve” effect related to the surgeon using first a technique and then the other, has not biased this study.

Similar to other studies, data regarding high frequency BC-PTA are lacking. This is a limitation, as higher frequencies are known to be more commonly affected by trauma during stapedotomy, regardless of the technique. Also, word recognition score would serve as a more sensitive indicator of potential cochlear injury after footplate fenestration, but this evaluation is not routinely performed at our Institution, and this is shared with other experiences [4].

## Conclusion

The present study is the first to compare hearing results and complications between CO_2_ fiber laser and microdrill in endoscopic stapes surgery. Our results demonstrated similar functional outcomes between the two groups, thus confirming endoscopic stapes surgery as a safe and effective technique, regardless of the instruments used.

## Data Availability

Data are available from the corresponding author upon reasonable request.
